# Aneuploidy: implications for protein homeostasis and disease

**DOI:** 10.1242/dmm.013391

**Published:** 2014-01

**Authors:** Ana B. Oromendia, Angelika Amon

**Affiliations:** David H. Koch Institute for Integrative Cancer Research, Department of Biology and Howard Hughes Medical Institute, Massachusetts Institute of Technology, Cambridge, MA 02139, USA.

**Keywords:** Aneuploidy, Disease, Protein folding

## Abstract

It has long been appreciated that aneuploidy – in which cells possess a karyotype that is not a multiple of the haploid complement – has a substantial impact on human health, but its effects at the subcellular level have only recently become a focus of investigation. Here, we summarize new findings characterizing the impact of aneuploidy on protein quality control. Because aneuploidy has been associated with many diseases, foremost among them being cancer, and has also been linked to aging, we also offer our perspective on whether and how the effects of aneuploidy on protein quality control could contribute to these conditions. We argue that acquiring a deeper understanding of the relationship between aneuploidy, disease and aging could lead to the development of new anti-cancer and anti-aging treatments.

## Introduction

Aneuploidy results in an ‘unbalanced’ genome in which chromosome(s), or pieces of chromosomes, are missing or supernumerary. The condition has a profound impact on cellular and organismal fitness (reviewed in Torres et al., 2008; Williams and Amon, 2009). In humans, aneuploidy is the leading cause of mental retardation and spontaneous abortions, and is a key characteristic of cancer, with an estimated 70–90% of all human solid tumors harboring aneuploid genomes (see Box 1 for a glossary of terms) (Weaver and Cleveland, 2006; Duijf and Benezra, 2013). Recent studies have also unveiled a connection between aneuploidy and aging, specifically that increases in aneuploidy are associated with premature aging and reduced lifespan (Baker et al., 2004; Baker et al., 2008; Wijshake et al., 2012; Baker et al., 2013).

Box 1. **Glossary**

**Aneuploid:** chromosome content that is not a whole multiple of the haploid complement; imbalanced karyotype.

**Centrosome:** cellular organelle from which microtubules nucleate.

**Euploid:** cognate chromosome content; balanced karyotype.

**Gene copy number:** number of times a gene is present in a genome.

**Gene dosage compensation:** the alteration of mRNA or protein expression to compensate for variation in DNA copy number.

**Heat shock response:** transcriptional response elicited by an increase in temperature.

**Proteotoxicity:** impediment in cellular function caused by aberrant or misfolded proteins.

**Spindle assembly checkpoint:** surveillance mechanism that halts cell cycle progression until all chromosomes have attached to the spindle.

**Unfolded protein response:** transcriptional response that results from misfolded proteins in the endoplasmic reticulum.

Systematic analyses of aneuploid yeast, mouse and human cells, and studies on cancer cell lines, suggest that aneuploidy causes chromosome-specific effects that are elicited by the increased (or decreased) number of copies of individual genes and/or combinations of a small number of genes present on the aneuploid chromosome (Tang and Amon, 2013). Changes in the gene copy number of regulators of gene expression lead to further disruption of cellular function. Studies in aneuploid budding yeast, mammalian cell lines, flies and plants have revealed a suite of phenotypes that are representative of aneuploidy. These phenotypes include a cell cycle delay in G1 (Torres et al., 2007; Stingele et al., 2012; Thorburn et al., 2013), metabolic alterations (Williams et al., 2008; Li et al., 2010), genomic instability (Sheltzer et al., 2011; Zhu et al., 2012) and proteotoxicity (Torres et al., 2007; Tang et al., 2011; Oromendia et al., 2012; Stingele et al., 2012). Furthermore, most aneuploid cells studied to date exhibit a transcriptional signature associated with slow growth and stress (Torres et al., 2007; Sheltzer et al., 2012; Stingele et al., 2012; Foijer et al., 2013). In *Drosophila*, activation of the stress kinase Jun N-terminal kinase (JNK) occurs in epithelial cells upon chromosome mis-segregation (Dekanty et al., 2012). Similarly, in mammals, chromosome mis-segregation causes activation of the stress kinase p38 and the stress-induced transcription factor p53 (Li et al., 2010; Thompson and Compton, 2010; Janssen and Medema, 2011). Understanding the origins of these phenotypes is important because this could provide insights into how chromosome mis-segregation and the resulting imbalanced karyotype impacts normal cell physiology and disease states. Here, we will give our perspective on one of the most striking aneuploidy-associated phenotypes: proteotoxicity; that is to say, a state in which the protein quality-control machinery of the cell (protein chaperones, autophagy or the ubiquitin proteasome system) is non-functional or overwhelmed, resulting in misfolded proteins. We will first summarize how aneuploidy causes proteotoxic stress and then discuss how this aspect of aneuploidy could contribute to human diseases and the aging process.

## Aneuploidy results in an unbalanced proteome that causes proteotoxic stress

The unbalanced genome caused by aneuploidy has been shown to translate into an unbalanced proteome – that is to say that the changes in gene dosage for the most part result in equivalent changes in protein levels (e.g. an extra copy of a gene results in twice as much protein). Studies of *Saccharomyces cerevisiae* aneuploid strains show that the abundance of ~80% of proteins changes in proportion to gene copy number (Pavelka et al., 2010; Torres et al., 2010). Interestingly, many of the proteins for which this is not true are subunits of multimeric complexes (Torres et al., 2007; Torres et al., 2010). Indeed, usually, subunits that are endogenously expressed in excess because of aneuploidy retain stoichiometric proportions within multimeric complexes (Torres et al., 2007; Torres et al., 2010). Stingele and colleagues looked at the relationship between gene dosage and protein levels in human aneuploid cells (Stingele et al., 2012). Analysis of the transcriptome and proteome of aneuploid human cells generated by chromosome transfer showed that most genes are expressed in proportion to their copy number, and proteins are translated in strong correlation with the abundance of mRNA, resulting in a dramatic change in cellular protein composition with aneuploidy (Stingele et al., 2012). However, as in aneuploid yeast, human aneuploid cells were also found to maintain a subset of proteins (enriched for complex subunits) at stoichiometric levels even if gene copy number was altered. The regulatory mechanisms responsible for this correcting process have not been elucidated. Overall, these data suggest that, although some proteins are maintained at stoichiometric levels, there is no general whole-chromosome ‘gene-dosage compensation’ mechanism for autosomes in yeast and mammals, as has been observed for sex chromosomes. This might not be the case in all organisms, however. Aneuploid *Drosophila* S2 cells have been reported to experience dosage compensation at the transcriptional level by means of the male-specific lethal (MSL) complex and additional mechanisms that compensate for differences in non-autosomal chromosome copy number (Zhang et al., 2010). Further studies in *Drosophila* aneuploid cells are needed to determine the status of their proteome.

**Fig. 1.**
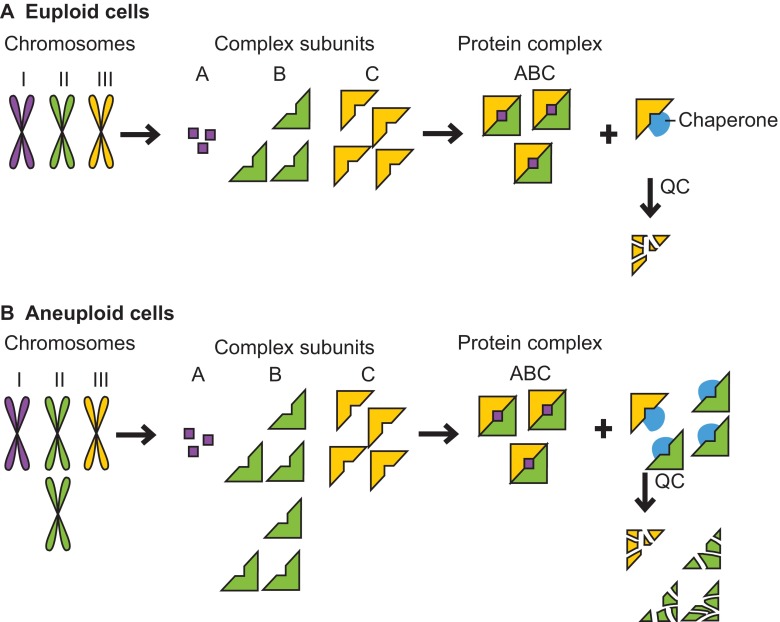
**Aneuploidy causes proteotoxic stress.** (A) Cells use protein quality-control (QC) and feedback mechanisms to maintain subunit stoichiometries of complexes whose subunits are encoded by different chromosomes. The protein quality-control machinery ensures accurate folding and maintains complex subunits that lack a binding partner in a soluble state. Eventually, excess and misfolded subunits must be degraded, as illustrated here by the yellow subunit that has been produced in relative excess. (B) Changes in chromosome number in aneuploid cells (shown here as disomy of the green chromosome) lead to a genomic imbalance that results in stoichiometric protein imbalances. Every subunit encoded by an unbalanced chromosome that functions in a protein complex lacks its binding partner(s) and must rely on cellular chaperones to maintain solubility and, if no binding partner is found, on the cellular proteases for its eventual degradation. This can lead to an increased burden on the protein quality-control systems and the exhaustion of the cellular protein quality-control machinery.

A key question resulting from the profound effects of aneuploidy on cellular protein composition is whether the simultaneous changes of the relative ratios of many proteins impacts upon the protein quality-control pathways of the cell. Chaperones and the degradation machinery – the 26S proteasome, proteases and autophagy – ensure that all proteins acquire their native conformation and prevent cellular toxicity by reducing the number of inappropriate interactions between proteins. In aneuploid cells, these protein quality-control systems must not only attend to the excess proteins produced from additional chromosomes, they must also support all excess subunits of complexes that are not in stoichiometric ratios with their binding partners (Fig. 1). Many protein-complex subunits are unstable unless bound to their partners, and will often bind to cellular chaperones to remain soluble until they have formed the complex (Boulon et al., 2010). Several previous studies have suggested that aneuploidy places strain on protein quality-control systems. As mentioned above, budding yeast, mouse and human aneuploid cells exhibit expression of cellular stress response genes (Torres et al., 2007; Sheltzer et al., 2012; Stingele et al., 2012), and this response includes upregulation of protein chaperones (Sheltzer et al., 2012). Human aneuploidies generated by chromosome transfer *in vitro* were found to have a transcriptional stress signature that shows upregulation of lysosome-mediated degradation and p62-dependent autophagy (Stingele et al., 2012; Stingele et al., 2013). Furthermore, many haploid *S. cerevisiae* strains harboring an additional chromosome (disomic yeast strains) were found to be sensitive to chemical compounds that impair protein quality control; many disomic yeast strains are sensitive to the proteasome inhibitor MG132, the ribosome poison cycloheximide, and the Hsp90 inhibitors radicicol and geldanamycin (Torres et al., 2007). Mouse embryonic fibroblasts (MEFs) trisomic for any of chromosomes 1, 13, 16 or 19 are more sensitive to the Hsp90 inhibitor 17-AAG than are wild-type MEFs (Tang et al., 2011). These results can be interpreted as follows: the aneuploid state causes proteotoxic stress, leading aneuploid cells to rely more heavily on their protein quality-control machinery. Thus, impairing chaperone function via use of chemical chaperone inhibitors is more detrimental to cells that are aneuploid than to cells that carry the appropriate number of chromosomes (euploid cells).

**Fig. 2.**
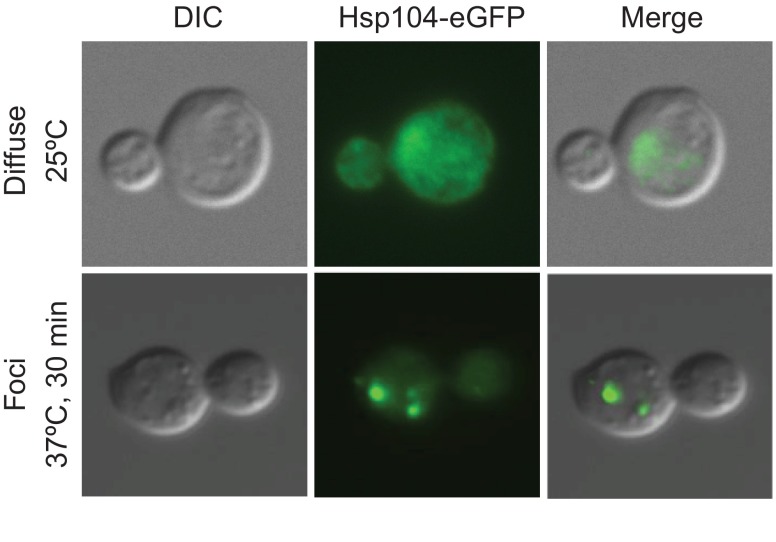
**Protein aggregates marked by Hsp104.** Example of Hsp104 foci that form in budding yeast cells, imaged by differential interference contrast (DIC) and fluorescence microscopy, upon increasing protein misfolding. Wild-type budding yeast cells carrying the disaggregating protein chaperone Hsp104 fused to eGFP were imaged while growing at 25°C in rich medium (YPD) and after a 30-minute heat shock, which induces protein misfolding, at 37°C. Hsp104-eGFP is diffusely localized throughout the cell under normal growth conditions, but heat-shocked cells accumulate protein aggregates that are seen as Hsp104–eGFP foci.

These observations prompted a direct test of the idea that aneuploidy and the ensuing protein imbalances lead to proteotoxic stress. Using Hsp104 fused to GFP as a marker for protein aggregation, it was shown that, irrespective of the chromosome identity or the method by which the aneuploidy was generated, aneuploid budding yeast strains are more likely to form protein aggregates than are euploid cells (Oromendia et al., 2012) (Fig. 2). This observation indicates impairment in protein quality control, either during the folding and/or disaggregation processes governed by chaperones and/or at the level of protein degradation. We will first discuss the effects of aneuploidy on protein chaperones and then summarize the effects of aneuploidy on proteasomal degradation.

### The impact of aneuploidy on chaperone systems

Protein chaperones are intricately involved in the maturation of a protein – from a polypeptide exiting the ribosome to acquiring the appropriate three-dimensional structure, to assembly into the appropriate complexes. Chaperones have been classified into several families according to their type of enzymatic activity, the co-chaperones they require and the clients that they aid in folding (Hartl et al., 2011). One initial study assessed the impact of aneuploidy on the ubiquitous chaperone Hsp90 in budding yeast. Hsp90 is a highly conserved chaperone that is essential for the survival of eukaryotic cells. It has been shown to have a limited repertoire of protein clients, most of which are kinases and signal transduction proteins (McClellan et al., 2007; Franzosa et al., 2011). Using assays that monitor Hsp90 activity *in vivo*, it was shown that 8 out of 11 disomes (haploid + one chromosome) tested showed reduced Hsp90 folding activity (Oromendia et al., 2012). This was surprising given that Hsp90 has been shown to be highly abundant in yeast, representing 1–2% of total protein (Borkovich et al., 1989; Neckers, 2007). It is not yet known why Hsp90 activity is limiting in so many different disomic yeast strains. It has been proposed that the presence of excess client proteins depletes the Hsp90 reservoir; however, *in vitro* folding assays are needed to exclude the possibility of a reduction in the folding activity of the Hsp90 molecules (Oromendia et al., 2012). It is unlikely that Hsp90 is the only chaperone system that is limiting in aneuploid yeast strains. We hypothesize that, when analyzed, most chaperone families will be compromised in at least some aneuploid cells. Different aneuploidies will, of course, affect the various chaperone families differently depending on the proportion of clients for a particular chaperone affected by a given aneuploidy.

It might seem surprising that the cell’s protein quality-control pathways cannot compensate for the presence of a single additional chromosome, which, depending on chromosome size, results in 2–12% of the yeast genome being imbalanced. However, even a 0.1% increase in misfolded proteins places a burden on the cell’s protein quality-control systems and leads to reduced cellular fitness, as judged by reduced doubling times in affected cells (Geiler-Samerotte et al., 2011). Crucially, the number of misfolded proteins requiring assistance from the cell’s protein quality-control pathways is increased in aneuploid cells. It is well established that many subunits of protein complexes only acquire a stable conformation by binding to other subunits of the complex. Thus, every single polypeptide produced by genes located on aneuploid chromosomes that normally has a binding partner is – in disomes – in excess. These polypeptides are then continuous clients of chaperones and prevent them from assisting other folding reactions. This interferes with the chaperones’ essential functions of mediating folding of essential proteins.

Interestingly, the number of aneuploid cells harboring aggregates decreases when the ratio of uncomplexed proteins to properly complexed proteins is decreased. By increasing base ploidy, i.e. by assessing the effects of aneuploidy in diploid rather than haploid strains, proteotoxicity was found to be lowered (Oromendia et al., 2012). This finding shows that proteotoxicity in aneuploid cells is to a significant degree the result of the protein stoichiometry imbalances. Of course, each extra chromosome can also encode proteins that absolutely require chaperones for their function, such as protein kinases and WD40-repeat proteins. The excess of these proteins presumably also places an additional burden on the cell’s protein quality-control systems. Thus, genomic imbalance caused by aneuploidy, even involving small chromosomes and few genes, can have a major impact on cellular protein quality control.

It is important to note that, although aneuploid yeast and human cells are able to elicit both a heat shock response and an unfolded protein response when prompted by heat stress or endoplasmic reticulum (ER) stress, they do not elicit these responses under normal (unchallenged) growth conditions (Oromendia et al., 2012; Stingele et al., 2012). This indicates that there is a proteotoxicity threshold required to elicit the acute heat shock or unfolded protein response that aneuploid cells do not meet. In contrast, MEFs that are trisomic for various different chromosomes constitutively upregulate Hsp72, an inducible member of the Hsp70 chaperone family (Tang et al., 2011). In summary, aneuploidy has a dramatic impact on proteome composition and, by extension, on the protein quality-control machinery of the cell.

### Effects of aneuploidy on protein degradation

In order to cope with alterations in protein homeostasis, cells degrade misfolded protein subunits by means of the 26S proteasome or by autophagy. Previous studies uncovered a nonsense mutation in the gene encoding the deubiquitylating enzyme Ubp6 as conferring improved proliferation (faster growth rate) properties to a subset of disomic yeast strains (Torres et al., 2010), implicating proteasome function in contributing to protein homeostasis in aneuploid cells. Ubp6 associates with the proteasome, where it removes ubiquitin chains from substrates and, in this function, not only plays a crucial role in ubiquitin recycling but also in regulating proteasomal degradation. In cells lacking *UBP6*, a number of proteasome substrates are degraded more rapidly (Crosas et al., 2006; Hanna et al., 2007; Torres et al., 2010; Sakata et al., 2011). Deletion of *UBP6* results in improved growth in a subset of disomes at standard growth temperatures (Torres et al., 2010) and of virtually all disomes analyzed at high temperature, when protein quality-control systems are even more challenged (Stacie E. Dodgson, personal communication). Importantly, deleting *UBP6* also attenuates the effects of disomy on protein composition by increasing the degradation of highly expressed proteasome substrates (Torres et al., 2010) and reduces protein aggregate formation (Oromendia et al., 2012). These findings lead to the intriguing notion that protein aggregation is, in part, responsible for the fact that aneuploid cells proliferate slower than their euploid counterparts.

Protein degradation is not only mediated by the proteasome: cells additionally deploy autophagy as a means of protein quality control (Kubota, 2009). Misfolded proteins are sequestered into aggregates and, in a p62-dependent manner, are targeted for autophagy. Autophagy can be monitored by assessing the number of light chain 3 (LC3)-positive foci, or by assaying the abundance of LC3-II, the autophagosome-specific lipidated form of LC3. Aneuploid human cell lines created by chromosome transfer and trisomic MEFs harbor significantly increased numbers of LC3 foci and LC3-II protein levels relative to the euploid control lines (Stingele et al., 2012). p62 protein abundance is also significantly increased in aneuploid human cell lines, suggesting an increase in p62-dependent autophagy in aneuploid human cell lines (Stingele et al., 2012). Because the effects on autophagy were detected in many aneuploid cell lines irrespective of the chromosomes that were supernumerary, higher levels of autophagosomes seems to be a cellular response to aneuploidy in mammalian cell lines, most likely deployed to manage the increased abundance of misfolded proteins.

In summary, proteotoxic stress and the cellular response to this stress are now recognized as important features of aneuploidy from yeast to human cells. We will discuss in the next section how this aspect of aneuploidy could contribute to human diseases.

## Aneuploidy and diseases of protein folding: lessons from Down syndrome

The connection between aneuploidy and disease has been at the forefront of the study of aneuploidy. Aneuploidy is extremely prevalent in solid tumors, with 70–90% estimated to have an unbalanced karyotype (Weaver and Cleveland, 2006; Duijf and Benezra, 2013). Cancer cells have also long been considered ‘chaperone addicted’ (Neckers and Neckers, 2002) and Hsp90 inhibitors are currently being developed as frontline chemotherapeutics (Wagner et al., 2013). The dependency of tumors on chaperones has been attributed to the need to efficiently fold oncogene products, which are often kinases and thus Hsp90 clients. However, the high levels of aneuploidy in cancer cells, and the proteotoxic stress that stems from such aneuploidy, could provide an additional explanation for their chaperone addiction. Further investigation of compounds that increase chaperone burden or that inhibit the function of chaperones might lead to the discovery of new cancer therapeutics with efficacy in a broad spectrum of human tumors.

The high degree of aneuploidy observed in cancers also begs the question of whether cancer cells have evolved mechanisms that allow them to tolerate high levels of karyotypic imbalances. One aneuploidy-tolerating mutation seems to be loss of p53 function. In normal cells, chromosome mis-segregation leads to activation of the tumor suppressor p53; the mechanisms whereby this occurs are still being elucidated and might be caused by multiple aspects of chromosome mis-segregation (Li et al., 2010; Thompson and Compton, 2010; Janssen and Medema, 2011). Generating a comprehensive list of genetic alterations that ameliorate the effects of aneuploidy and their characterization will shed light on tumor evolution. It will allow us to address important questions such as when such mutations arise in response to aneuploidy, and whether and how they contribute to tumorigenesis. Compounds that neutralize aneuploidy-tolerating mutations could also provide new avenues of cancer treatment.

In addition to cancer, autosomal aneuploidy has been associated with numerous human conditions that result in impaired development. In humans, three viable trisomies have been described. An additional copy of chromosome 21 leads to Down syndrome, chromosome 18 to Edward’s syndrome and a trisomy of chromosome 13 to Patau syndrome. Of these, only Down syndrome individuals survive past childhood. It will be interesting to determine whether protein quality-control systems are affected in individuals with these constitutional aneuploidies. Chromosome 21 harbors the fewest genes of all human chromosomes and might thus not cause a significant burden on the cellular protein quality-control pathways. Determining the contribution of impaired protein homeostasis to the pleiotropic phenotypes of this syndrome could nevertheless be warranted because Down syndrome is strongly associated with a protein-folding disease. Individuals with Down syndrome are predisposed to early-onset Alzheimer’s disease (AD). Although the main cause of AD in Down syndrome individuals is likely to be the additional copy of the *APP* gene encoded by chromosome 21 (reviewed in Kingsbury et al., 2006), mice overexpressing *APP* (which encodes amyloid beta A4 protein) do not fully recapitulate all the AD-like phenotypes seen in Down syndrome mouse models (Cataldo et al., 2003). Conversely, mouse models of Down syndrome that lack the *APP* gene still exhibit some of the AD-like pathologies (Table 1), suggesting that duplication of the *APP* gene might not be the only cause of early-onset AD in Down syndrome individuals.

**Table 1.**
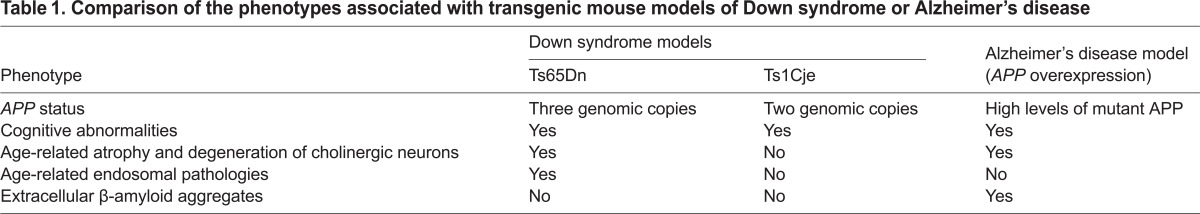
Comparison of the phenotypes associated with transgenic mouse models of Down syndrome or Alzheimer’s disease

The two mouse models of Down syndrome are Ts65Dn and Ts1Cje (Table 1). Ts65Dn mice are trisomic for the distal region of chromosome 16 (92 genes homologous to human chromosome 21 from *APP* to *MX1*); this segment contains nearly two-thirds of the human chromosome 21 homologous genes, including the Down syndrome critical region (DSCR) and the *APP* gene. Ts65Dn mice are also trisomic for a segment of mouse chromosome 17 (60 genes) that is non-homologous to genes on human chromosome 21. Ts1Cje mice are trisomic for a smaller region of chromosome 16 that includes the DSCR but not *APP* (67 genes homologous to chromosome 21, from *SOD1* to *MX1*; approximately two-thirds of the trisomic region of Ts65Dn mice), and they are monosomic for the telomeric region of mouse chromosome 12 (seven genes) (Cataldo et al., 2003). In addition to these two mouse models of Down syndrome, transgenic mice have been generated that harbor an additional copy of a mutant form of *APP* (K670M/N671L) that has been identified in a Swedish family with early-onset AD (‘*APP* overexpression’, Table 1). Although many of the phenotypes are shared between the mice, an increased copy of *APP* is not sufficient to recapitulate all of the AD-related phenotypes of Down syndrome mouse models. Thus, perhaps a reduced ability to maintain protein homeostasis contributes to the AD pathology in individuals with Down syndrome.

In addition to the constitutive aneuploidies of chromosomes 13, 18 and 21, mutations in genes encoding the spindle assembly checkpoint component BUBR1 or centrosome components have been shown to lead to mosaic variegated aneuploidy (MVA), a disease characterized by aneuploidies showing a random widespread distribution in the body (Hanks et al., 2004; Snape et al., 2011). There are no published evaluations of proteotoxicity in MVA cell lines, but, given that protein quality-control systems have also been shown to be impaired in complexly aneuploid yeast strains (haploid strains that are aneuploid for more than one chromosome) (Oromendia et al., 2012), it would be of interest to investigate whether the same is true in the case of individuals with MVA and to determine how this contributes to the disease phenotype.

There is evidence to suggest that aneuploidy might be associated with a range of neurodegenerative diseases such as AD, Parkinson’s disease, amyotrophic lateral sclerosis (ALS), spinocerebellar ataxia and Huntington’s disease. Neurodegenerative diseases are all characterized by the misfolding and aggregation of specific proteins. Intriguingly, aneuploid yeast strains were found to be more prone than wild-type strains to form aggregates of a hard-to-fold protein containing a polyQ stretch, which is also considered a model for Huntington’s disease. Expressing this polyQ protein also impairs proliferation of aneuploid yeast strains more than that of euploid controls, indicating that expression of a hard-to-fold protein affects the fitness of aneuploid cells (Oromendia et al., 2012). Could aneuploidy be a contributor to neurodegenerative protein-folding diseases? Several studies have suggested that as many as 30% of embryonic neurons and 15–20% of adult neurons harbor aneuploidies (Rehen et al., 2001; Rehen et al., 2005; Yurov et al., 2005; Yurov et al., 2007). Why aneuploidy would be more prevalent in neurons compared with cells of other tissues is unclear, but it would provide an intriguing explanation for the prevalence of protein-folding diseases in this cell type. Future studies and additional methods to assess aneuploidy in tissues will be necessary to assess the degree and types of aneuploidy comprehensively in the brain and to determine the effects of aneuploidy on neurodegenerative diseases.

## Aneuploidy and aging

All organisms age, and this process is characterized by, among other phenotypes, the following: genomic instability, epigenetic alterations, deregulated nutrient sensing, mitochondrial dysfunction and loss of proteostasis (reviewed in López-Otín et al., 2013). Furthermore, aging is the primary risk factor for major human diseases, including cancer, diabetes, cardiovascular disorders and neurodegenerative pathologies. Interestingly, recent studies by van Deursen and co-workers have provided intriguing links between aneuploidy and the aging process. They found that mice carrying hypomorphic alleles in the spindle assembly checkpoint gene *BUBR1*, which also serves as a mouse model for MVA, harbor high levels of aneuploidy (Baker et al., 2004). Remarkably, these animals age prematurely. Mice carrying hypomorphic alleles of *BUBR1* prematurely develop phenotypes characteristic of old age, such as cataracts, sarcopenia, growth retardation, muscle wasting, fat loss and cardiac arrhythmias (Baker et al., 2004; Wijshake et al., 2012; Baker et al., 2013). Intriguingly, overexpression of *BUBR1* has the opposite effects – it leads to a reduction in chromosome mis-segregation and hence reduced aneuploidy (Baker et al., 2013), and the animals live longer and have a longer life without ailments. Furthermore, cardiac function is increased, and muscle and renal atrophy and glomerulosclerosis are reduced (Baker et al., 2013). Exactly how aneuploidy might result in aging remains to be determined, but we propose that the systemic impacts of aneuploidy on cell physiology, such as proteotoxicity, as discussed here, together with metabolic changes and genomic instability, are the source of aneuploidy-induced aging. It will be very interesting to determine whether mutations that suppress the adverse effects of aneuploidy also delay aging and extend the span and quality of life.

## Concluding remarks and future perspectives

Aneuploidy has a profound impact on most, if not all, cellular functions. This Review has centered on the consequences of aneuploidy on the protein quality-control mechanisms of the cell and the implications this could have on our understanding of human diseases and aging. Aneuploidy has been shown to cause proteotoxic stress in yeast and mammalian cells. Irrespective of the identity of the supernumerary (or deleted) chromosomes, aneuploidy triggers proteotoxicity, and thus it is a phenotype inherent to the aneuploid state itself. Understanding the full impact of this condition on cells and organisms will not only deepen our knowledge of the consequences of an imbalanced karyotype but will provide fundamental insights into developmental disabilities such as Down syndrome and diseases such as cancer. Exciting too is the possibility that it might also unveil the mysteries of aging.
